# A Pulmonary Pleomorphic Carcinoma Patient with Exudative Retinal Detachment Secondary to Choroid Metastasis as Initial Presentation—A Case Report

**DOI:** 10.3390/medicina57060539

**Published:** 2021-05-28

**Authors:** Kathy-Ming Feng, Yi-Hao Chen, Jiann-Torng Chen, Li-Fan Lin, Wen-Chiuan Tsai, Ching-Long Chen

**Affiliations:** 1 National Defense Medical Center, Department of Ophthalmology, Tri-Service General Hospital, Taipei City 114, Taiwan; fengk57@gmail.com (K.-M.F.); doc30879@mail.ndmctsgh.edu.tw (Y.-H.C.); jt66chen@gmail.com (J.-T.C.); 2 National Defense Medical Center, Department of Nuclear Medicine, Tri-Service General Hospital, Taipei City 114, Taiwan; fanlin2@gmail.com; 3 National Defense Medical Center, Department of Pathology, Tri-Service General Hospital, Taipei City 114, Taiwan; drtsaiwenchuan@mail2000.com.tw

**Keywords:** exudative retinal detachment, choroid metastasis, pleomorphic carcinoma

## Abstract

Choroid metastasis is the initial presentation of pleomorphic carcinoma (PC) of the lung. PC is classified as poorly differentiated non-small cell lung carcinoma. It has a tendency to metastasize early and has a poor response to chemotherapy, which often results in poor prognosis. We report the case of a 63-year-old woman with a one-month history of deteriorating vision in the left eye. Fundus examination, fluorescein angiography, indocyanine green angiography, and B-scan sonography demonstrated choroidal metastasis of the left eye. Positron emission tomography/computed tomography (PET/CT) revealed a tumor with increased uptake in the left upper lung. Subsequent bronchoscopic biopsy confirmed a pleomorphic carcinoma of the lungs. Choroid metastasis as an initial presentation of PC in the lung is rare. Usually, it represents the late course of disseminated disease with hematogenous spread. Prompt diagnosis is imperative for patients to immediately initiate treatment.

## 1. Introduction

The choroid is a common site for the occurrence of metastases from breast cancer in women and lung cancer in men because of its high vascular supply [1]. Patients with choroid metastasis experience decreased visual acuity, attributed to exudative retinal detachment (ERD); however, in 15–20% of patients, the condition remains asymptomatic until it is accidentally discovered during a routine examination [2,3]. The disruption of the blood-retinal barriers in ERD could be attributed to inflammation, infection, and neoplastic and vascular pathological conditions [4]. According to the theory of the pathophysiology of metastasis, tumor cells migrate from the primary site through the circulatory or the lymphatic system and are retained in a new organ that they encounter [3]. Hence, once a patient is diagnosed with ERD secondary to choroidal metastasis, a full medical history and physical examination for any systemic involvement is crucial for definitive diagnosis of the tumor sites. A review reported that the histopathologic type of most lung cancers with uveal metastases was non-small cell lung cancer (NSCLC) [5]. Pulmonary pleomorphic carcinoma (PC) is classified as a poorly differentiated NSCLC, which is highly malignant and resistant to chemotherapy and radiotherapy. It accounts for only 0.3% of all lung cancer cases [6]. There have been reports of distant metastasis to the brain, bone, breast, adrenal gland, esophagus, jejunum, rectum, and kidney [7,8]; however, ocular metastasis has not been reported to date. Herein, we report a case of PC of the lung with multiple metastases presenting with ERD secondary to choroidal metastasis. Ascertaining the secondary cause of early ERD is crucial for planning the treatment strategy.

## 2. Case Report

**Figure 1:**
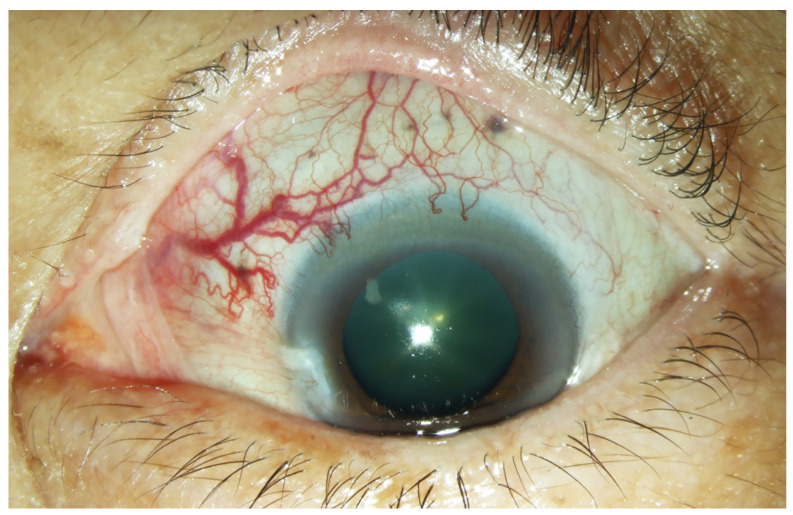
External photograph of the left eye. Prominent dilated episcleral vessels in the superonasal region of the left eye.

A 63-year-old woman presented with progressively decreased vision in the left eye over a period of one month. Ophthalmological examination revealed a visual acuity of 6/12 in the right eye and 6/60 in the left eye. Dilated and tortuous episcleral vessels along with pterygium were prominent in the left eye (Figure 1).

**Figure 2:**
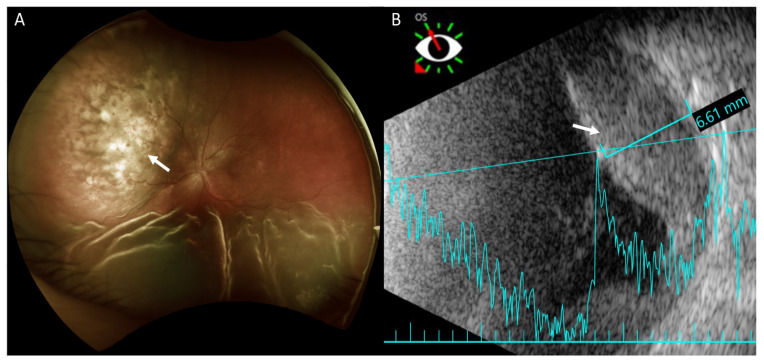
Fundus photography and B-scan ultrasonography of the left eye. (**A**) Fundus photography shows exudative retinal detachment in the inferior retina and an amelanotic choroidal mass in the superior quadrant (arrow) in marked relief. Exudative retinal detachment is seen mainly on the sides of the choroidal mass. (**B**) Ultrasonography shows the achromic mass in the superior region as a hyperechoic lesion in the subretinal region (arrow) with a thickness of approximately 6.61 mm.

Fundus examination revealed retinal detachment mainly in the inferior region and an amelanotic choroidal mass with retinal detachment in the superior quadrant of her left eye (Figure 2A). Ultrasonography showed that this achromic choroidal mass was a hyperechoic lesion in the subretinal region with a thickness of approximately 6.61 mm, raising the possibility of intraocular tumor or metastasis (Figure 2B).

**Figure 3:**
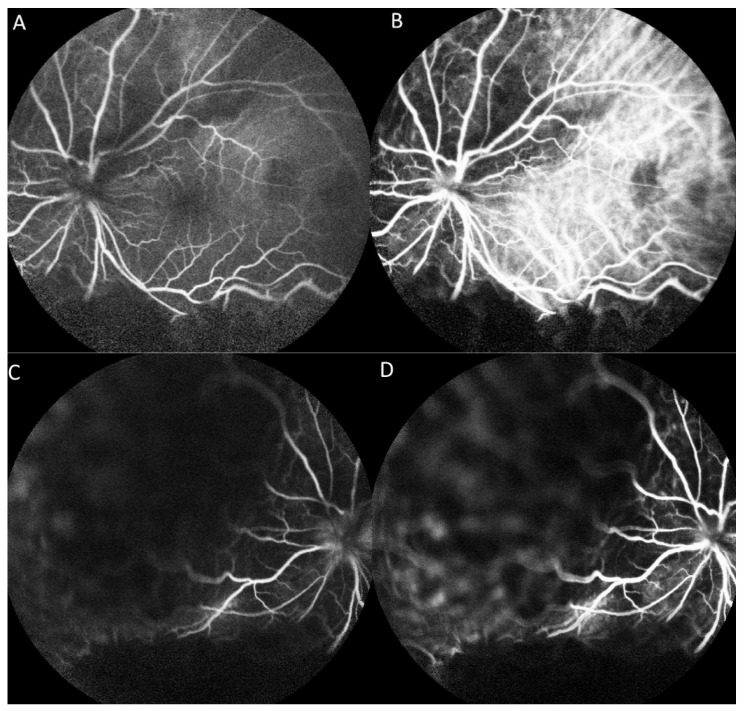
Fluorescence angiography (FA) and indocyanine green angiography (ICG) of the left eye. (**A**) FA shows hypofluorescence in the superior and inferior regions and two hypofluorescent points in the temporal region of the fovea in the early phase. (**B**) ICG shows a hypofluorescent region corresponding with that on FA in the early phase. (**C**) FA shows hypofluorescence in the superior and inferior regions in the late phase. (**D**) ICG shows hypofluorescent regions corresponding with those on FA in the late phase.

On fluorescein angiography (FA) and indocyanine green angiography (ICG), we observed hypofluorescence throughout the phases in the superior and inferior regions of the left eye. As there was only one mass on the fundus, this suggested that the hypofluorescence was partly related to the choroidal mass and partly to the ERD. Additionally, two hypofluorescent spots were noted in the temporal region of the fovea (Figure 3). These two small hypofluorescent spots were barely visible as orange-colored lesions on fundus examination (Figure 2A) and exhibited hypofluorescence throughout the angiography. These could be small choroidal metastatic lesions. The presence of these multifocal lesions supported the diagnosis of choroidal metastasis rather than choroidal melanoma or choroidal angioma, which are almost always unifocal.

**Figure 4:**
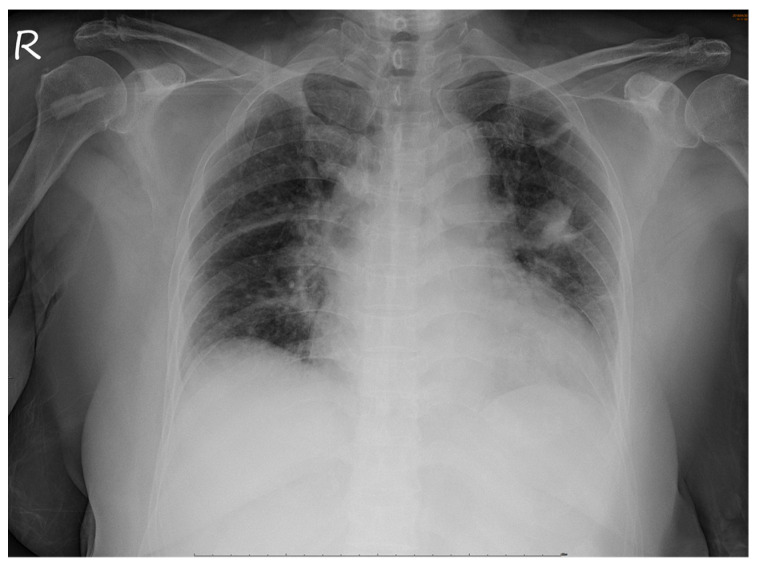
Chest radiograph. A non-homogenous patch is seen in the superior lobe of the left lung.

No abnormalities were noted in the right eye. Serological tests for infection, autoimmune diseases, and tumor markers were negative. However, chest radiography revealed a non-homogenous opacity in the superior lobe of the left lung (Figure 4).

**Figure 5:**
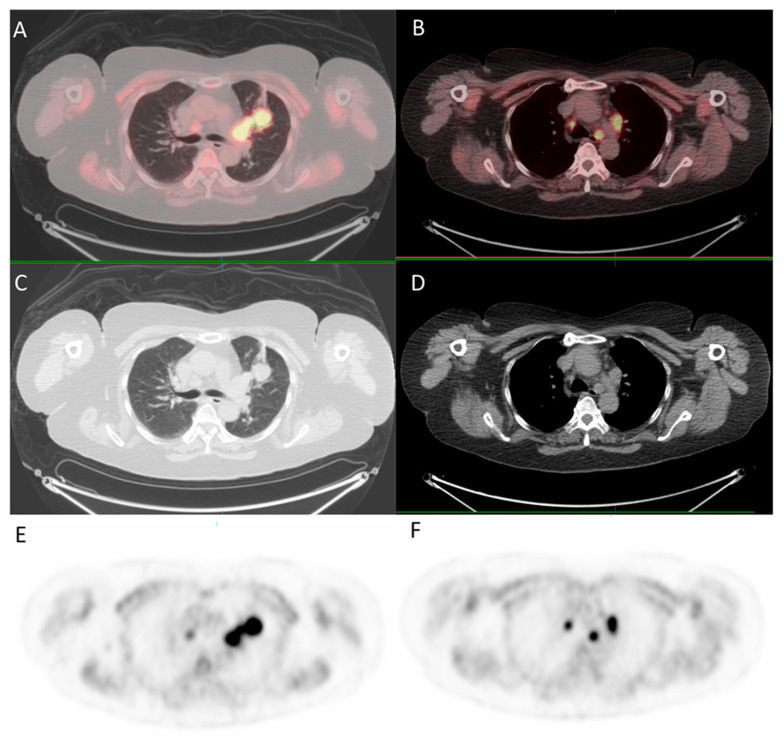
^18^F-fludeoxyglucose positron emission tomography/computed tomography (^18^F-FDG PET/CT) for whole-body evaluation. (**A**,**B**) axial fused PET/CT showing focal uptake of the lung and mediastinum, respectively; (**C**,**D**) axial CT; (**E**,**F**) PET.

**Figure 6:**
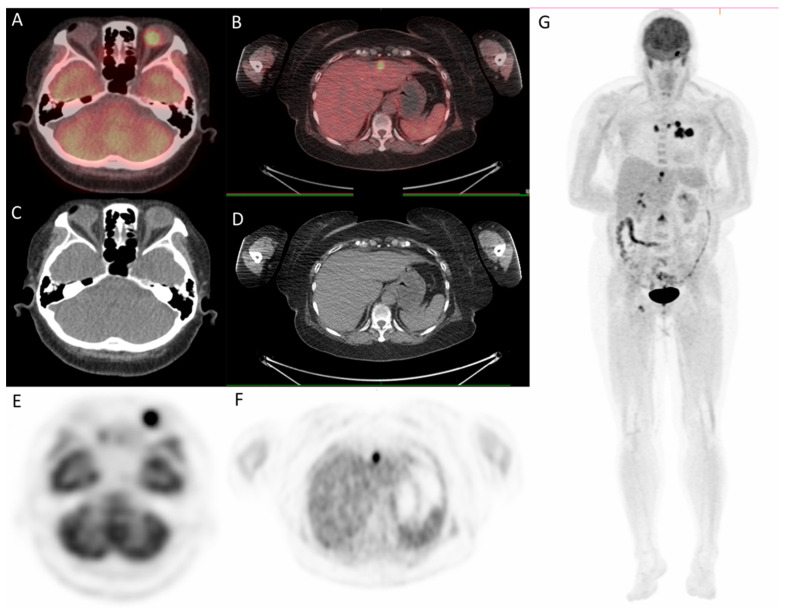
^18^F-fludeoxyglucose positron emission tomography/computed tomography (^18^F-FDG PET/CT) for whole-body evaluation. (**A**,**B**) axial fused PET/CT showing focal uptake of left eye and liver, respectively; (**C**,**D**) axial CT; (**E**–**G**) PET. PET/CT.

Since the patient had a family history of lung cancer, whole-body positron emission tomography/computed tomography (PET/CT) was suggested; the results showed focal uptake in the left eye with a standardized uptake value (SUV) of 10.8, left upper lung (SUV: 11.5), mediastinum (SUV: 14.8), liver (SUV: 9.6), and bone (SUV: 10) (Figure 5 and Figure 6).

**Figure 7:**
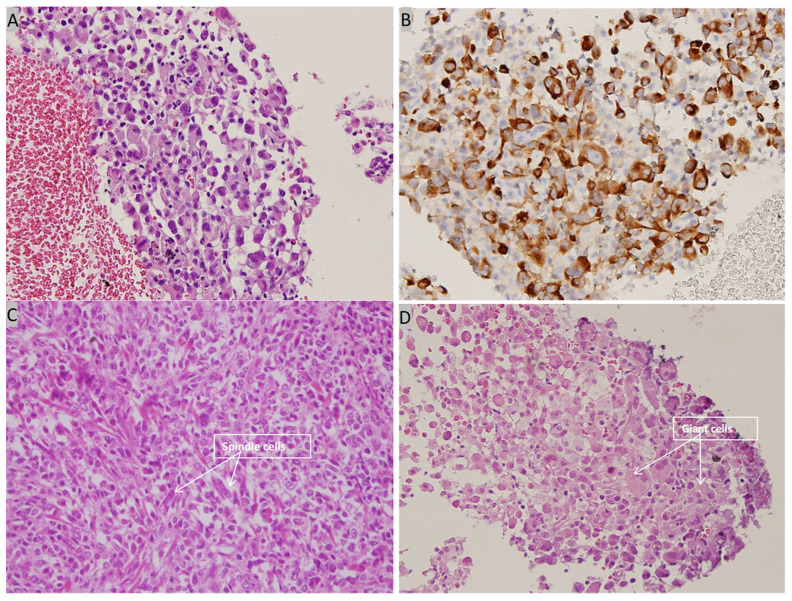
Histopathological examination of bronchoscopic biopsy. (**A**) High power view showing many tumor cells with eccentric nuclei and eosinophilic cytoplasm (H&E, 400×). (**B**) Immunohistochemical staining is positive for cytokeratin (cytokeratin, 400×). (**C**) High power view showing spindle cells with pleomorphic nuclei (H&E, 400×) (**D**) High power view showing giant cells (H&E, 400×).

Contrast-enhanced CT of the lung revealed a 2.8-cm tumor in the upper lobe of the left lung with regional lymph node metastasis. Abdominal sonography revealed fatty infiltration in the liver parenchyma, obscuring the focal lesion. Spine radiography revealed spondylosis of the lumbar spine with marginal spur formation. Giant cells and spindle cells were noted on transbronchial biopsy, and immunohistochemistry studies revealed that tumor cells were positive for vimentin and cytokeratin (Figure 7). The final diagnosis was stage 4 PC of the lung with nodal and multiple distant metastases, and the patient was transferred to the oncology department for further medical management. After referral to the oncology department, the patient was informed about her cancer and local radiotherapy of the left eye combined with systemic chemotherapy/immunotherapy was suggested. Because the patient chose hospice care at home, these treatment strategies were not implemented, and the patient died 4 months after the diagnosis.

## 3. Discussion

Choroid metastasis is a common intraocular finding in malignancies in adults because of its abundant vascular supply. A 63-year-old woman presented with loss of vision in the left eye, and ophthalmic evaluation revealed ERD secondary to a choroid tumor. PET/CT showed multiple metastases, and bronchoscopic biopsy revealed PC of the lung. To the best of our knowledge, there are no reports of choroid metastasis of PC of the lungs.

Symptoms of choroid metastasis include decreased vision, flashes, and floaters, which are often caused by foveal ERD [1]. Shields et al. [9] found that ocular pain is higher in metastatic lung cancer than in breast cancer, and Shah et al. reported that 14% of 194 uveal metastatic patients presented with ocular pain [5]. It was postulated that the pain could be due to scleral invasion. Our patient presented with blurred vision as the sole symptom of ERD.

Examinations to support the diagnosis of choroidal metastasis include fundus examination, ultrasonography, FA, ICG, and optical coherence tomography [2]. Furthermore, magnetic resonance imaging of the brain and orbit can rule out any coexisting metastatic brain lesions. On fundus examination, choroid metastases usually appear as solid, spotted, and yellow lesions [10], similar to those observed in the superonasal region in our patient (Figure 2A). Ultrasonography provides the depth and dimensions of the lesion; however, the clinical appearance of choroid metastasis or primary tumors such as melanoma or hemangioma can be similar. Thus, investigating the growth pattern and histoarchitecture of choroid tumors is important, with metastatic tumors being more lobulated and having higher reflectivity than primary choroid melanomas [3]. It was difficult to determine if the choroid tumor was lobulated in our patient because the hyperechoic lesion was situated beneath the ERD. However, the lesion showed high reflectivity. Choroid tumors usually have a similar pattern on FA, with a hypofluorescent pattern in the early phase and heterogeneous hyperfluorescence in the late phase. In addition, pinpoint hyperfluorescence may occur [3]. Thus, it is difficult to confirm if the lesion is a choroid tumor, such as melanoma or choroid metastasis using FA. In our patient, because of ERD, we did not observe a heterogeneously hyperfluorescent pattern in the late phase. ICG could potentially help differentiate choroidal tumors. Early rapid filling with extreme hyperfluorescence within 1 min, and sometimes a late wash-out phenomenon is often present in choroidal hemangioma [11,12]. Choroidal melanoma has varied presentation but usually demonstrates slower filling and less hyperfluorescent intensity compared to choroidal hemangiomas. Visible intrinsic vascularization is seen in 66% of choroidal melanomas, but most are hypofluorescent throughout [12]. Choroidal metastasis usually appears hypofluorescent in all phases [3,12], as seen in our patient.

An achromic choroidal mass can be a choroidal hemangioma (diffuse or circumscribed), achromic melanoma, or choroidal metastasis. It is important to note that approximately 67% of patients presenting with choroidal metastasis have already been diagnosed with primary cancer; however, in 18% of the patients, the primary tumor is diagnosed later [9,13]. Surprisingly, the primary cancer site is not detected in 17% of the patients even after systemic evaluation. Therefore, ophthalmologists have a major role in the diagnosis of cancer [9,13].

Lung cancer is the most common primary tumor for choroid metastasis in men [9], and the frequency of ocular metastasis in lung cancer in postmortem ocular examination is 6.1% [14]. Therefore, loss of vision due to lung cancer is extremely rare and usually indicates the final stage of the disseminated disease. The incidence of lung cancer has increased steadily over the years, and lung cancer has become the leading cause of cancer-related deaths worldwide. Most lung cancers are either NSCLC or small-cell lung cancer. PC is considered a poorly differentiated NSCLC, with an incidence of approximately 0.1–0.4% of all lung cancers [15]. It consists of spindle and/or giant cells, as seen within the biopsy of our patient. The incidence of PC is higher in elderly men, and cigarette smoking is considered a risk factor [7]; nevertheless, our patient did not have any history of smoking. PC grows rapidly and immediately invades adjacent structures, as shown by its aggressive clinical course and tendency to metastasize early [15]. Distant metastasis can occur in the brain, bone, breast, adrenal gland, esophagus, jejunum, rectum, and kidneys [7,8]. In our patient, the biopsy revealed pleomorphic carcinoma of the lung, and multiple metastases were observed on PET/CT imaging.

In the last decade, conventional imaging modalities, such as plain radiography, ultrasound, CT, and magnetic resonance imaging, have been widely used to provide clear structural images of the tumor [16]. PET, which uses short-lived radioisotopes, such as fluorine-18 to label glucose, can detect malignancy and be used for staging, restaging, or monitoring treatment responses [17]. PET combined with CT provides simultaneously high sensitivity of tracer distribution and high precision of localization, facilitating the detection of tumor sites in patients with choroid metastasis. In the present case, PET/CT scan was utilized early for detection of tumor sites, and it highlighted multiple uptakes in the left eye, left upper lung, mediastinum, liver, and bone. In addition to providing useful clues to the possible primary tumor sites, it was fast and efficient.

One of the limitations of our study was that a fine-needle aspiration biopsy was not performed for the left eye; hence, the definite diagnosis of choroidal metastasis from primary lung cancer is questionable. However, because of the aggressive nature of PC, with a tendency to metastasize early and the findings of clinical examination of the choroidal lesion, the possibility of choroidal metastasis from the lung is highly likely. Another limitation was that the patient refused the suggested treatments and chose hospice care at home. Therefore, we did not obtain any information about the treatment or the evolution of the lesion under treatment.

## 4. Conclusions

In conclusion, choroid metastasis from PC of the lung is rare, and careful examination of the primary tumor site is essential. PET/CT is a useful tool to assist in the detection of other tumor sites in choroid metastasis. Early detection of these metastatic lesions would benefit the patient and clinicians in formulating appropriate treatment strategies, as well as avoiding any futile interventions, thereby reducing costs in the long term.

## Data Availability

The data supporting the conclusions of this article are included within the article.
